# Recent advances in the application of polysaccharides to enhance the functional properties and extend the shelf life of milk proteins: A review

**DOI:** 10.1016/j.fochx.2026.103584

**Published:** 2026-01-23

**Authors:** Mohamed Aamer Abubaker, Duoduo Zhang, Haorui Ma, Runru Xiang, Yiran Wang, Abdalla Musa Elimam, Majida Al-Wraikat, Yongfeng Liu

**Affiliations:** a College of Food Engineering and Nutritional Science, Shaanxi Normal University, Xi'an 710119, China; b Department of Biology, Faculty of Education, University of Khartoum, Khartoum 11111, Sudan; c Department of Biology, Faculty of Science, Al-Baha University, Al Baha 75311, Saudi Arabia

**Keywords:** Polysaccharides, Milk proteins, Functional food development, Dairy product optimization

## Abstract

Milk proteins are vital to dairy products, providing essential functional properties like thickening, gelling, and emulsification, along with high nutritional value. However, their stability, sensitivity to environmental conditions, and texture control pose challenges. Polysaccharides enhance milk protein functionality by improving texture, stability, and nutritional quality through mechanisms such as co-solubility, phase separation, and complex coacervation. The strength of protein-polysaccharide interactions depends on factors like pH, ionic strength, and processing conditions. Polysaccharides stabilize protein structures, reduce denaturation, and improve texture retention. This knowledge aids in developing dairy products that meet the growing demand for healthier, sustainable, and high-quality foods. Moreover, polysaccharides, particularly those from natural sources, contribute to sustainability by reducing waste and promoting biodegradable packaging. Future research on optimizing polysaccharide-protein interactions could further enhance the functionality and nutritional benefits of dairy products, catering to health-conscious and environmentally aware consumers.

## Highlights

• Polysaccharides enhance milk protein functionality, supporting clean-label dairy.
• Recent polysaccharide techniques improve milk protein functions and stability.
• Focus on mechanisms enhancing milk protein texture, emulsification, and gelation.
• Polysaccharides support sustainability by reducing waste and enabling eco-packaging.
• Aimed at advancing high-quality dairy products that meet consumer demands.

## 1 Introduction

For a long time, dairy products have been considered essential components of the human diet (Yuzbashian et al., 2022). Global milk output rose by 0.5% in 2017, according to the Food and Agriculture Organization (FAO), and is predicted to climb by 22% by 2027. The United Nations Food and Agricultural Organization (UNFAO) reports that for the production of fresh milk are camel milk (0.5%), goat milk (2%), sheep milk (1%), buffalo milk (15%), and cow milk (81%) (Zhang et al., 2024). One of the main meals in a person's diet is milk, which has a variety of nutrients. High-quality proteins found in milk include serum, whey protein, and casein, which make up around 80% of the total amount of milk proteins and are not found in other foods (Ravash et al., 2022). These proteins perform a range of vital functions in food products, including emulsification, thickening, gelling, and foaming. Specifically, casein and whey proteins are widely utilized in food products due to their nutritional benefits, functional properties, and capacity to create diverse textures (Nayik et al., 2024). However, despite their versatility, these proteins have several functional limitations that can impact their performance in food processing and product development (Shang et al., 2023). One major challenge is their sensitivity to environmental factors, which can significantly alter their functionality. For example, high temperatures, such as those encountered during pasteurization or cooking, can denature whey proteins, leading to undesirable aggregation and texture issues in protein-enriched beverages (Wang, et al., 2024). Furthermore, milk proteins become unstable in acidic environments, often precipitating at low pH levels, which results in phase separation or overly firm gels in products like yogurt and cheese (Ahmadi et al., 2024). Similarly, in high-salt environments, such as cheese brines, protein aggregation reduces solubility and functionality. Caseins, which constitute 80% of milk proteins, exist in micellar structures that are relatively insoluble in water, particularly at extreme pH levels, limiting their use in clear protein beverages (Runthala et al., 2023). Although whey proteins are more soluble, they too can aggregate under heat or in the presence of salts and acids, leading to sedimentation in protein-fortified drinks. Furthermore, milk proteins exhibit weak emulsification in low-pH conditions, causing phase separation and compromising the stability of emulsions, especially under environmental stresses (Nayik et al., 2024).

Therefore, recently researchers have found that natural products such as polyphenols, flavonoids, and polysaccharides offer promising solutions to the challenges encountered by milk proteins in food processing (Pang et al., 2025). These compounds exhibit stabilizing properties that can improve the functional performance of milk proteins under various environmental stresses. Among them, polysaccharides are particularly versatile and effective in enhancing the overall quality, texture, and shelf life of milk proteins (Ding et al., 2025). Their multifunctionality, including water retention, gelation, emulsification, and neutral flavor, makes them especially valuable for incorporation into milk proteins processing, providing comprehensive benefits that extend beyond just dairy applications. Moreover, Wang et al. (2025) found that the addition of polysaccharides can significantly alter the functionality of whey protein, particularly by enhancing its gelling and rheological properties. As two ubiquitous biopolymers, proteins and polysaccharides frequently coexist in the same food chain. The thickening, shape, processing stability, and texture retention of food ingredients are significantly influenced by their interactions (Li, Lai, et al., 2024). As natural substances, polysaccharides are essential for maintaining the integrity of milk proteins (Ding et al., 2025). Polysaccharides from various sources show biodegradable, biocompatible and non-toxic features which make a suitable polymer in several filed like medicine, and food (Kalpana Manivannan et al., 2024). Because of their non-toxic, widely available, reproducible, and health-promoting qualities, they have become more and more popular as food additives to enhance and/or change the texture, flavor, character, and nutrition of food (Wang, et al., 2024). With the variety of polysaccharide sources, such as plants, fungi, and algae, used in dairy products, β-glucans from oats and barley are particularly noted for their technological roles, including gel formation and moisture retention (Alabdallah & Alluqmani, 2022). Additionally, β-glucans derived from yeast and mushrooms show potential for enhancing the functional properties of dairy products (Buniowska-olejnik, 2022).

This study explores the role of natural polysaccharides in enhancing milk protein functionality. It highlights how polysaccharides improve texture, stability, and quality in dairy products, enhancing emulsification, water retention, and gelation. The review provides insights into using polysaccharides to create healthier, more resilient dairy products that meet modern consumer demands for nutrition and quality.

## 2 Milk proteins structure and their biological functions

Milk proteins are the primary source of its well-known health benefits, valued for both their nutritional and biological properties (Pratelli et al., 2024). There are two main types of proteins in milk: caseins and whey proteins. These proteins work together to give the body amino acids, bioactive peptides, and the ability to bind minerals, all of which help with growth, immunological defense, and general health.

### 2.1 Structure of milk proteins

Casein, a type of heteroprotein, is the main protein in milk. It has many subunits, such as αs1-, αs2-, β-, and κ-caseins, which come together to form micelles that are held together by calcium phosphate nanoclusters. These micelles are important for the storage and movement of minerals, and they also change the viscosity and whiteness of milk (Muñoz-Salinas et al., 2022).Whey proteins, on the other hand, are a combination of globular proteins that stay soluble even after casein precipitation. The main parts are α-lactalbumin (α-LA) and β-lactoglobulin (β-LG), as well as serum albumin and immunoglobulins (Inagaki et al., 2024). These proteins have specific tertiary structures that let them bind to minerals, vitamins, and tiny ligands, which is what gives them their many biological functions.

### 2.2 Species-specific variations in protein composition

The structural organization of caseins and whey proteins is preserved; however, their relative quantities differ among species. Caseins make up about 80–85% of the total protein fraction in ruminant milks such cow, goat, sheep, and buffalo (Casertano et al., 2021). On the other hand, non-ruminant animals like camels and donkeys make milk that has more whey proteins 50–60%, which makes donkey milk very similar to human milk in this way (Cimmino et al., 2023). Sheep and buffalo milk have the most protein overall 12–16% and 9–12%, respectively, while camel milk usually has 3.5–4.5% protein and a more balanced whey-to-casein ratio. These changes between species affect how well milk proteins can be digested, how likely they are to cause allergies, and how well they work, but they don't change the basic structure of milk proteins.

### 2.3 Biological functions of milk proteins

Caseins serve as a slow-digesting protein source and play a structural role in delivering minerals. They are rich in calcium and phosphorus, forming stable calcium phosphate complexes essential for skeletal development and bone strength. Magnesium, associated with caseins, further supports muscle contraction and enzymatic activity. Whey proteins, on the other hand, are rapidly digested and provide bioactive peptides with antioxidant, antimicrobial, and immunomodulatory activities. They are also linked with key minerals such as potassium and sodium, which regulate fluid balance and nerve conduction, as well as zinc and iron, which contribute to immune defense, wound healing, and oxygen transport (Saubenova et al., 2024).

Beyond their nutritional and mineral-binding functions, milk proteins contain diverse bioactive peptide sequences that display strong biological activity (Al-Wraikat et al., 2024). For example, peptides derived from goat milk casein hydrolysates have demonstrated significant antibacterial effects, effectively suppressing *Staphylococcus aureus* and *Escherichia coli* (Ningsih et al., 2024). Together, casein and whey proteins not only enhance the nutritional value of milk but also contribute to its therapeutic and functional potential. Strengthening the roles and health benefits of milk proteins is therefore crucial for improving product quality, meeting dietary demands, and increasing competitiveness in the food and nutrition market. The biological activities, bioactive peptides, and conditions linked to milk proteins from different species are compiled in the Table 1 below.

**Table 1:** Some biological functions related to milk proteins from various species.

Source	Milk protein	Conditions	Biological function	References
Camels	Milk protein fractions (whey + casein fractions)	Different extraction methods; fraction comparison	α-Amylase inhibitory activity (antidiabetic) ranged 19.10 ± 1.40 to 97.40 ± 1.50%, varying by fraction/method.	Harizi et al. (2024)
Goat	α-lactoglobulin, β-lactalbumin, β-casein, α-s1-casein, α-s2-casein, and κ -casein	Gastrointestinal digestion in silico using chymotrypsin, trypsin, and pepsin	83 possible antimicrobial peptides (AMPs) were predicted to be released; 13 of these peptides were validated.	Sansi et al. (2022)
Zhongdian Yak (ZY)	Whey proteins (RNA helicase (typically a soluble, globular protein) and uncharacterized protein (A0A3Q1LFQ2)	Molecular docking, Simulated Gastrointestinal Digestion (SGID)	Immunoprotective action; the production of bioactive peptides, including ACE inhibitory and antioxidant peptides.	Li, Li, et al. (2024)
Donkey	Bioactive peptides (FDM peptides)	RAW264.7 macrophage model; LPS-stimulation; NO measurement	FDM peptides reduced nitric oxide (NO) production in LPS-stimulated RAW264.7 cells, showing anti-inflammatory potential.	Ning et al. (2025)
Cow	Lactoferrin	Purification of LF from bovine milk	LF investigated as a candidate bioactive protein contributing to antidiabetic properties of milk.	Khan et al. (2022)
Sheep	α-s1, α-s2, β-casein, κ-Cn, α-lactalbumin, β-lactoglobulin	Using pepsin, trypsin, chymotrypsin, and enzyme combinations for in silico gastrointestinal digestion	A promising source of peptides that lower blood pressure and prevent diabetes.	Iram et al. (2022)
Binglangjiang buffalo & Dehong buffalo	Whey-derived peptides (12 predicted bioactive peptides)	In silico bioactivity prediction following peptide identification	12 peptides predicted to have immunomodulatory, anti-inflammatory, and antimicrobial activities.	Zhao (2025)

## 3 Milk proteins: challenges and the role of natural additives

According to the FAO report, world milk production grew by 1.1% in 2024, reaching approximately 950 million tons (Gupta & Dubey, 2025). During the same period, the global population increased, and per capita milk consumption also rose. This growth in dairy consumption, coupled with the overall rise in food demand, contributes to the ongoing challenges in environmental sustainability (Corigliano et al., 2025). Given their bio-polymeric nature, milk proteins are being increasingly investigated for their potential applications across various sectors of the food industry. However, despite their promising attributes, milk proteins face several significant challenges that may compromise their functionality and quality (Stastna, 2024). Enzymatic degradation, whether from naturally occurring or microbial enzymes, can lead to undesirable alterations in flavor and texture. Additionally, microbial contamination during production, processing, or storage poses considerable risks to both safety and shelf life. Furthermore, heat-induced protein denaturation alters the functional characteristics of milk proteins, negatively impacting their ability to gel and emulsify, thus limiting their effectiveness in food applications (Hu et al., 2024). By breaking down lipids and proteins, oxidative stress exacerbates these problems by causing rancidity and a reduction in nutritional value. Additionally, allergenic sensitivities to particular milk proteins pose problems for consumer safety and product development. Also, variations in quality may result from the nutritional variety of milk proteins, which are impacted by the diet and breed of the animal (Bi et al., 2024).

**Fig. 1:**
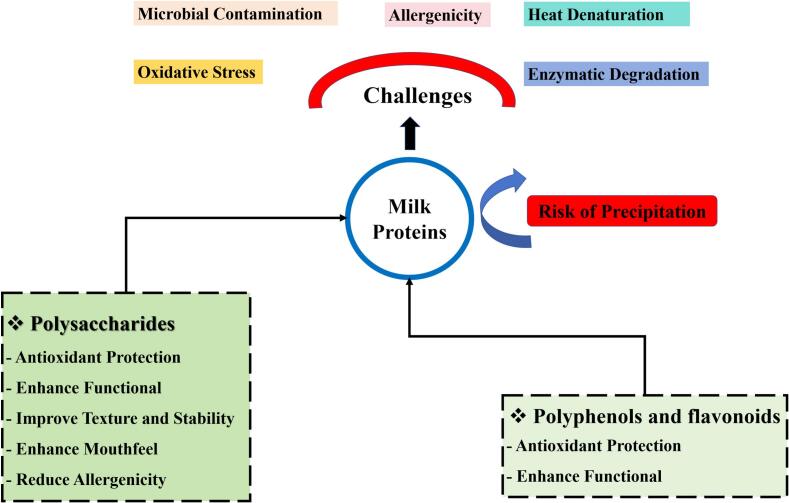
Challenges and the role of natural additives.

Therefore, the addition of natural substances such polyphenols, flavonoids and polysaccharides offers a potential remedy for the problems milk proteins encounter as showed in Fig. 1 (Jia et al., 2022). Polyphenols improve milk proteins' antioxidant properties and maintain milk's nutritional value. They enhance protein functionality, boosting emulsification and gel formation. However, high polyphenol concentrations can negatively affect proteins, causing precipitation or reduced solubility (Mao et al., 2024). Flavonoids can contribute to improved flavor and appealing color in dairy products, and both flavonoids and other polyphenols are widely valued for their antioxidant potential. Polysaccharides, meanwhile, are often used to enhance mouthfeel, texture, and overall product stability and are generally compatible with maintaining milk protein solubility under typical formulation conditions (Xue et al., 2024).

## 4 Polysaccharides and milk protein interactions

### 4.1 Polysaccharides sources, structure, and functions

**Fig. 2:**
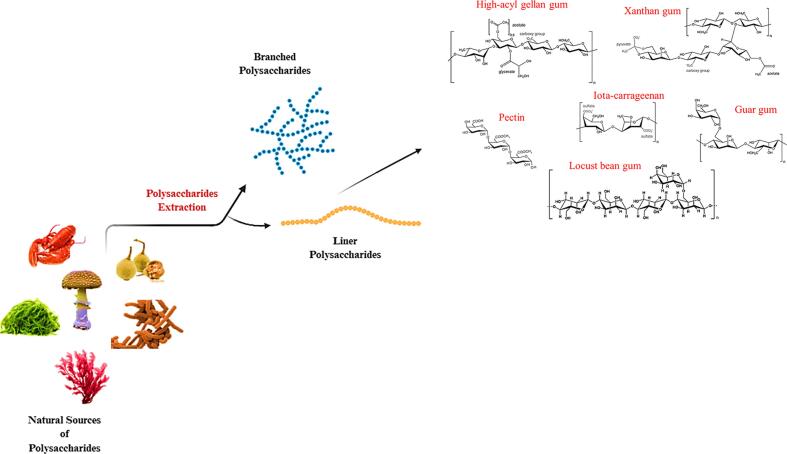
Polysaccharide structures from various natural sources.

Polysaccharides are complex carbohydrates composed of monosaccharide units linked by glycosidic bonds. The specific types of monosaccharides they contain and the nature of the glycosidic bonds determine their structural characteristics (Xu et al., 2025). Polysaccharides can be derived from various natural sources, including plants, bacteria, and algae, with each source requiring a distinct extraction and purification process as shown in Fig. 2.

Based on their monosaccharides composition, polysaccharides are classified into homopolysaccharides and heteropolysaccharides. Homopolysaccharides, such as cellulose and starch, consist of a single type of monosaccharide, whereas heteropolysaccharides, such as guar gum and pectin, contain a mixture of different monosaccharides (Song et al., 2024). Polysaccharides contain active functional groups like -OH, -COOH, -COCH3, and -NH2, which enable them to interact effectively with proteins (Mahmoudi et al., 2024). These groups influence polysaccharide solubility, reactivity, and functionality, making them useful for protein interactions in applications such as emulsification and encapsulation. Food-grade polysaccharides, approved for use in food formulations, enhance texture by leveraging their physicochemical properties hydration, gelation, emulsification, and thickening which are closely linked to their structural characteristics. These properties make them vital in improving food texture and stability.

### 4.2 Methods for preparing polysaccharide-milk protein complex

**Fig. 3:**
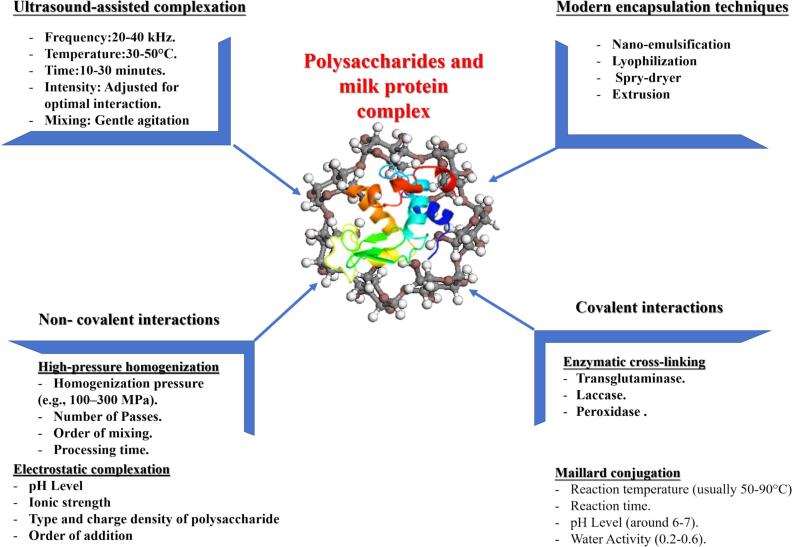
Polysaccharides-milk protein complex preparation methods.

The development of polysaccharide-milk protein complexes has gained considerable attention in recent years due to their ability to enhance food texture, stability, and overall functional properties. These complexes offer improvements such as better texture, increased nutritional bioavailability, and prolonged shelf life, making them highly valuable for a variety of food applications (Bourouis et al., 2023). Numerous sophisticated preparation methods have been developed to facilitate and regulate polysaccharide-protein complex formation, achieving the benefits mentioned earlier Fig. 3. These include both covalent strategies, such as enzymatic cross-linking and Maillard conjugation, and non-covalent methods like electrostatic complexation and high-pressure homogenization. Heat-induced complexation enhances protein denaturation and interactions with polysaccharides, improving emulsifying, foaming properties, and stability. Ultrasound-assisted complexation uses high-frequency sound waves to enhance interactions, preserving bioactive compounds without excessive heat (Han, Zhu, et al., 2024). Together, these methods provide versatile approaches for optimizing the functional properties of polysaccharide-protein complexes (Kan et al., 2021). Every approach has distinct benefits and can be modified to give the final complexes particular characteristics. Furthermore, to improve stability, safeguard bioactive chemicals, and regulate nutrient release, contemporary encapsulation techniques like extrusion, spray drying, freeze drying, and nano-emulsification are also used (Zabot et al., 2022). Building on that, Li, Yang, et al. (2023) found that the functional, structural, and rheological characteristics of whey protein isolate (WPI) after extrusion pretreatment (E-WPI) are greatly improved by the addition of inulin polysaccharides, especially at 15%. Water-holding capacity, emulsifying activity, emulsion stability, foaming ability, and foaming stability all showed significant improvements; the foaming ability increased by up to 162.97% when compared to unextruded WPI. Rheological investigation verified increased apparent viscosity and pseudoplastic fluid behavior in all samples, while FTIR spectroscopy showed a rise in β-turn structures and hydrogen bonding interactions between WPI and inulin. Additionally, Giles et al. (2025) explored the use of co-spray drying to improve interactions between whey protein and guar gum, xanthan gum, and maltodextrin, thereby enhancing the sensory properties of whey protein for older adults. Furthermore, Li, Liu, et al. (2023) discovered that employing polysaccharides from honeysuckle leaves (PHL) and whey protein isolate (WPI), nano-emulsification successfully produced a stable emulsion. This technique improved the interactions between PHL and WPI by creating tiny, stable droplets with a large surface area using ultra-sonication. The PHL-WPI combination was evenly distributed and the droplet size was decreased by ultrasound. A nano-emulsion with a median droplet size of 317.70 nm, near the target of 320.20 nm, was produced under ideal conditions. The PHL-WPI emulsion, in particular, showed improved stability for β-carotene against UV irradiation, demonstrating the potential of nano-emulsification to enhance the protective properties of WPI-based emulsions.

### 4.3 The main factors of polysaccharides-protein complex interaction

Polysaccharide-protein interactions significantly impact the stability, texture, and functionality of solutions, making them important in both biological systems and industrial food applications (Li et al., 2022). The nature of the interaction between proteins and polysaccharides in aqueous solutions varies depending on the specific conditions and characteristics of each molecule (Cortés-Morales et al., 2021). In some cases, when intermolecular interactions reach equilibrium, proteins and polysaccharides may coexist in the solution without strongly interacting, resulting in uniform distribution or co-solubility. This typically occurs in highly diluted solutions or when the complexes remain sufficiently soluble, allowing for homogeneous mixing (Ma et al., 2024). Protein-polysaccharide interactions can lead to behaviors like complex coacervation, depletion interactions, or thermodynamic incompatibility, influencing the mixture's physical structure. Factors such as molecular weight, charge density, hydration, and rheological properties affect viscosity, thermal stability, and functional properties, guiding the optimization of these complexes for various food system applications.

#### 4.3.1 Mixing behaviors

In an aqueous solution, protein-polysaccharide interactions can lead to four behaviors, including co-solubility, where both maintain individual structures and remain soluble. This results in a stable solution, reducing milk protein sedimentation through thickening, stabilization, encapsulation, depletion interactions, and improved interfacial properties (de Souza et al., 2023). For example, Silva et al. (2021) found that the maximum turbidity of protein-polysaccharide combinations correlates with the best yield, suggesting ideal interaction circumstances that support co-solubility while preserving the unique properties of the biopolymers. On the other hand, phase separation results from thermodynamic incompatibility when the two molecules interact negatively. Because of this incompatibility, the polysaccharide and protein separate into several domains, each of which is rich in a single component, resulting in a mixture that is heterogeneous (Yan et al., 2024), this was confirmed by Sarantis et al. (2021) found that When micellar casein and oat β-glycan interact negatively, thermodynamic incompatibility results. The two components separate into different domains due to this incompatibility, with amounts of 0.75–6% casein and 0.5–2% β-glycan. The mixture is heterogeneous because each domain is abundant in either the protein or the carbohydrate. The size and molecular weight of the polymers are important factors that affect thermodynamic incompatibility because larger polysaccharides may have more excluded volume effects and steric hindrance, which decreases their miscibility with proteins (Du et al., 2022). Another factor that affects compatibility depends on the pH and ionic conditions in the solution is electrostatic interactions, in which oppositely charged molecules attract and similarly charged molecules repel one another. Furthermore, hydrophobic contacts have an impact on separation, especially since hydrophobic proteins have a tendency to interact poorly with hydrophilic polysaccharides; warmth can further intensify hydrophobic interactions.

**Fig. 4:**
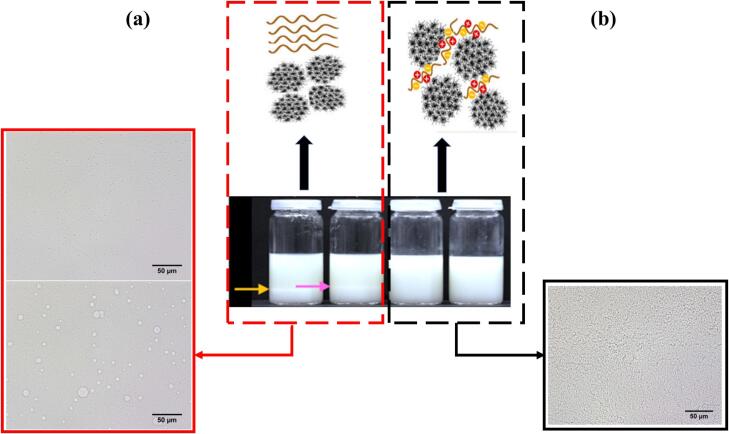
Polysacchraides-milk protein interaction mixing behavior: (a) Xanthan gum and guar gum interact with skimmed Heumilch, resulting in phase separation; (b) iota-carrageenan and gellan gum show no phase separation, as confirmed by microscopic images (Hege et al., 2020; Ma et al., 2024).

Another behavior, depletion interaction, involves an indirect attraction between protein molecules mediated by polysaccharides, where the presence of polysaccharides creates an osmotic pressure that brings proteins closer together, potentially resulting in aggregation or clustering. Hege et al. (2020) found that phase separation caused by depletion interaction was observed when xanthan gum and guar gum were added to milk-based beverages as shown in Fig. 4a. Casein micelles and other whey proteins stayed in the lower phase, whereas xanthan gum and other whey proteins accumulated in the upper phase. Casein micelles in the lower phase and guar gum in the top phase separated identically, indicating that depletion interactions might be the cause of this separation. These discoveries advance our knowledge of how milk proteins and polysaccharides interact, providing guidance for enhancing the rheological and sensory characteristics of beverages made with milk. Lastly, complex coacervation is driven by electrostatic attractions between oppositely charged proteins and polysaccharides, forming dense, coacervate-rich droplets containing high concentrations of both components, which separate from the surrounding solution (Zheng et al., 2025), and also Hege et al. (2020) discovered that when iota-carrageenan and gellan gum are dissolved in milk, respectively, no macroscopic phase separation is seen, and the data point to the development of complexes between the whey proteins and the hydrocolloid as illustrated in Fig. 4b.

In food and pharmaceutical applications, this coacervation is very helpful for stabilizing emulsions or encasing delicate molecules. The predominant phenomenon in each particular protein-polysaccharide system is determined by variables such as pH, ionic strength, and molecular characteristics, which collectively influence the mixture's stability and structure in aquatic settings.

#### 4.3.2 Physicochemical properties

The physicochemical characteristics of protein-polysaccharide complexes are another crucial factor to take into account (Li, Su, et al., 2023). This includes investigating how the molecular traits and structural alterations of polysaccharides and proteins affect their interactions and the system's overall features (Cortés-Morales et al., 2021). Since the size and charge distribution of proteins and polysaccharides greatly influence their interactions, molecular weight and charge density are essential variables. Stronger electrostatic contacts, which are especially important in complicated coacervation, can be encouraged by molecular weight and charge density size, which can affect the development and stability of coacervate or aggregated structures (Sabadini et al., 2024), according to that Nakamura et al. (2023) found that that the high molecular weight (∼1000 kDa) of both high methyl-esterified (HM-SPPS) and low methyl-esterified (LM-SPPS) pea polysaccharides contributes significantly to their stabilizing effects on milk proteins through steric repulsion. However, their differing degrees of methylation and resultant charge characteristics strongly influence their interactions with acidified milk proteins. LM-SPPS, with lower methylation, provides more negative charges, which enhances electrostatic repulsion between proteins, effectively stabilizing the protein dispersion by preventing aggregation. In contrast, HM-SPPS, with fewer charges due to higher methylation, leads to bridging flocculation through chain entanglements, as it lacks sufficient electrostatic repulsion to counter aggregation. Additionally, hydration and water-binding capacity play essential roles; the varying affinities of proteins and polysaccharides for water can alter the overall moisture retention of the system, which is vital for applications, impacting texture and juiciness (Flory & Alavi, 2024). Protein-polysaccharide interactions also alter the solution's or gel's viscoelasticity and rheological characteristics, which impacts food products' mechanical strength, gelation, and flow characteristics (Pan et al., 2024). In products where specific textures are essential, including yogurts, sauces, and dressings, these rheological characteristics are particularly significant. Furthermore, for applications requiring heat treatment, the gelation behavior and thermal durability of protein-polysaccharide complexes during heat processing are crucial (Igartúa et al., 2022).

#### 4.3.3 Functional implications

The features and uses of these systems are largely determined by the functional consequences of protein-polysaccharide interactions in addition to mixing behaviors. Stability and shelf life are important factors; the stability of emulsions, foams, and other colloidal systems is influenced by various mixing behaviors (Xue et al., 2024). Complex coacervation, for instance, can improve emulsion stability by creating a strong interfacial layer that shields proteins from oxidation and denaturation, thus increasing shelf life (Peng et al., 2024). Additionally, the nutritional value and sensory appeal of food products are influenced by the interactions between proteins and polysaccharides. While phase-separated systems brought on by thermodynamic incompatibility can provide grainy or undesired textures, co-soluble systems may produce smooth textures.

### 4.4 Functional and mechanistic effects of polysaccharides on improving milk protein

Improving the functions of milk proteins has important ramifications for nutrition, food science, medicine, and biotechnology, among other sectors. In a variety of dietary systems, polysaccharides are essential for improving the functional qualities of milk proteins (Shang et al., 2023). Adding polysaccharides, which interact with milk proteins to enhance foaming stabilization, emulsification properties, support gelation and texturization, increase solubility and water-holding capacity, and lengthen the shelf life of dairy products, is one of the most efficient ways to accomplish this. On the other hand, polysaccharides contribute not only by enhancing the dairy functions, but also through specific molecular mechanisms, including electrostatic interactions, hydrogen bonding, hydrophobic interactions, water-binding effects, viscosity enhancement, and covalent conjugation via the Maillard reaction (Babu et al., 2024).

#### 4.4.1 Foaming stabilization

Foaming milk is important for the taste and function of many foods and drinks, from coffee to dairy goods. The foaming ability, which is mostly determined by casein and whey proteins, affects the texture, mouthfeel, and quality of the product by stabilizing bubbles with their amphiphilic structures. These proteins include both hydrophilic and hydrophobic parts, which lets them quickly stick to the air-water interface and make viscoelastic films that lower surface tension, stop bubbles from coming together, and keep the foam structure. Caseins make thick, sticky layers around bubbles, while whey proteins stick to bubbles more quickly, which speeds up the formation of the first foam. Together, their opposite behaviors make sure that the foam stays stable. Polysaccharides improve this process in certain ways: they make the aqueous phase thicker, which slows down the flow of liquid between bubbles, and they interact with proteins through electrostatic or covalent bonds to make interfacial coatings stronger. These interactions cause changes in shape that make more hydrophobic sites available, which strengthens adsorption at the interface and makes the material more elastic, which helps the foam stay stable for longer. Linking this to the role of polysaccharides in foaming stabilization, the inclusion of polysaccharides, as observed in the referenced research, is critical in increasing foam structure and stability (Han, Zhu, et al., 2024). Wang et al. (2021) found that foam stability was significantly enhanced in the presence of trehalose and pectin during pasteurization. In line with this, Li, Wang, et al. (2024) explored that the foam stability of goat whey protein was significantly improved when conjugated with polysaccharides such as gum Arabic and citrus pectin during the Maillard reaction.

#### 4.4.2 Emulsification properties

**Fig. 5:**
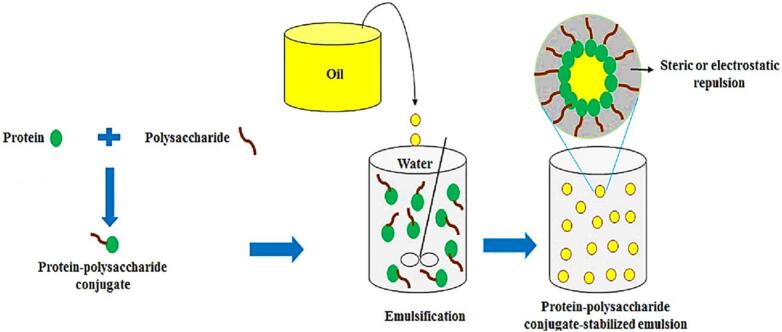
Polysaccharides-protein conjugate stabilized emulsion (Nooshkam et al., 2023).

Polysaccharides improve emulsification mainly because they can interact with proteins at the oil-water interface in several ways, such as by increasing viscosity, causing steric hindrance, creating electrostatic interactions, and forming covalent bonds. These interactions change the shape of proteins, expose hydrophobic groups, and make interfacial coatings thicker and more elastic. All of these things work together to keep droplets from coming together and forming larger droplets, which keeps the emulsion stable for longer. Polysaccharides are commonly employed to augment the emulsification characteristics of milk proteins, leveraging these mechanisms. As demonstrated in Fig. 5, the literature consistently shows that emulsions stabilized by protein polysaccharide conjugates are far more stable when stored than those stabilized by native proteins (Lv et al., 2024). Polysaccharides do not directly lower interfacial tension like typical emulsifiers do. Instead, they stabilize emulsions by changing the viscosity of the continuous phase and interacting with proteins and other surface-active agents. For example Ding et al. (2021) found that whey protein isolate (WPI) fragments, when covalently linked to malt dextrin through the Maillard reaction, exhibited strong emulsifying properties similar to soy protein isolate (SPI). Despite undergoing enzymatic hydrolysis with different enzymes, trypsin and alcalase, WPI remained molecularly well-dissolved in solution, which contributed to its stable emulsification performance. The degree of hydrolysis (DH) had a lesser impact on WPI compared to SPI, as WPI maintained a consistent emulsifying ability regardless of the enzyme used or hydrolysis level. These results suggest that WPI, due to its well-dissolved nature, shows robust emulsifying potential when combined with polysaccharides, making it a reliable choice for stabilizing emulsions. Similarly, Seo and Yoo (2021) demonstrated that the addition of κ-carrageenan (CG) to milk protein isolate (MPI) significantly improved its emulsifying stability. The conjugation of MPI with CG induced structural changes, confirmed by increased browning intensity, and led to larger fat droplets (D [3,2], D [4,3]) compared to the non-heated mixture. Specifically, droplet sizes increased from ∼1–2 μm to ∼1–4 μm as reaction time progressed. The emulsions stabilized by MPI-CG conjugates showed a significant increase in emulsion stability, as indicated by the emulsion stability index (ESI). This highlights the crucial role of polysaccharides like CG in enhancing emulsion stability by forming a thicker interfacial layer, improving both emulsifying and stabilizing properties of milk proteins. On the other hand, Li, Qiu, et al. (2024) discovered that the stability of processed cheese kept at room temperature was significantly increased when pectin was added to a WPI-stabilized fat emulsion to create a WPI–pectin bilayer. When compared to WPI alone, pectin greatly inhibited free oil release and decreased texture softening by strengthening the interfacial layer surrounding fat droplets and preventing coalescence and fat migration. Additionally, the bilayer system created a more homogeneous microstructure with smaller, more evenly distributed fat pools, demonstrating pectin's critical function in improving emulsion stability and oil retention throughout storage.

#### 4.4.3 Structural integrity and texture

Polysaccharides can improve the structure and texture of milk protein systems by affecting protein networks. Polysaccharides make the protein matrix stronger and improve its macroscopic qualities, like hardness, water-binding capacity, and creaminess, by changing the shape of the proteins, making them thicker, and trapping water. These mechanisms lower syneresis, encourage gel formation, and create a protein structure that is more cohesive and uniform, which improves mouthfeel and sensory appeal. In practice, polysaccharides create hydrated matrices in the protein network, which helps products like cheese and yogurt hold onto water better and keeps whey from separating***.*** In practice, mogrosides and locust bean gum polysaccharides form hydrated matrices within the protein network, which improves water retention and minimizes whey separation (syneresis) in products such as cheese and yogurt (Ban et al., 2023). This hydrated matrix mimics fat functionality, stabilizes emulsions, and ensures a uniform distribution of proteins and fat globules, critical for desirable texture and shelf-life stability (Leal et al., 2024) and (Xu et al., 2024). Polysaccharides fill in the spaces between protein networks on a microstructural level, stopping them from clumping together. This makes structures that are finer, denser, and more elastic, which makes it harder for them to separate into other phases. This kind of stability is especially helpful in dairy products that are high in protein, where polysaccharides keep things from clumping, and in goods that are lower in fat, where they make up for the loss of texture that comes with removing fat (Yao et al., 2024).

According to that, Wang et al. (2023) demonstrated that adding *Lycium barbarum* polysaccharide (LBP) and jujube polysaccharide (JP) to goat milk cheese compositions can have a major impact on the microstructural, rheological, and textural properties. A notable improvement in yield and rheological characteristics was achieved by adding 1% JP, which produced cheese with the maximum water-holding capacity, hardness, and a strong, dense casein network. On the other hand, cheeses enhanced with LBP had a softer texture, more moisture, and less fat.

#### 4.4.4 Gelation and texturization

Polysaccharides make it possible to improve the gelation and texturization of milk proteins, which is necessary to make the best dairy products. Polysaccharides change the structure of casein micelles and improve the texture of dairy products by generating functional complexes through electrostatic attraction, hydrogen bonding, and depletion interactions. The pH, temperature, and ionic strength of the contacts affect them, which makes it possible to make new microstructures and functional gels. Polysaccharides have a big effect on how protein networks form in milk by changing the links between milk proteins. This makes the gel more stable and structured. For example, Brüls-Gill et al. (2024) exhibited that through electrostatic forces and depletion mechanisms, polysaccharides interact with milk proteins to support their gelation and texturization. Polysaccharides like xanthan, guar gum, and γ-carrageenan facilitate the synthesis of protein networks more quickly during acid-induced milk gelation when the pH level is higher than when the gelation process begins. By altering the gel's viscosity and mechanical characteristics, polysaccharides help to improve the final texture of dairy products by accelerating the creation of protein networks and enhancing gelation and texturization. Mileti et al. (2024) shown that adding pectin to β-casein improves the formation of gelling by causing a solid-like structure to form over 3.5 h at concentrations of 1 and 10 g/L. As a result of this connection, a stable gel network develops, giving the protein matrix stability and mechanical strength. The results of the investigation showed that the interfacial shear trend indicates a strong stabilizing impact of pectin's derived from citrus, which intensifies with longer ageing times, guaranteeing a cohesive and durable gel structure in the finished product. On the other hand, the results obtained by Sarraf et al. (2023) found that adding xanthan gum (XG) to whey protein concentrate (WPC) improved texture consistency. The mechanical characteristics of the emulsion gel were enhanced by increasing the XG content, resulting in a more robust and even structure surrounding the oil droplets. In particular, the consistency value of the WPC emulsion gel increased from 11.48 N s to 19.96 N s at 0.6% concentration, indicating that XG successfully improved the texture by stabilizing and enhancing the gel.

#### 4.4.5 Mitigate the allergenicity of milk proteins

By modifying their structure, improving digestion, and hiding epitopes, polysaccharides can reduce the allergenicity of milk proteins (Sabaghi & Maleki, 2024). Polysaccharides physically bind to and cover the allergenic regions of milk proteins, making it impossible for the immune system to identify these epitopes. Furthermore, interactions between polysaccharides can change the secondary and tertiary structures of proteins, either reducing or eliminating the accessibility of the epitopes. Additionally, by improving protein digestion and increasing the proteins' susceptibility to enzymatic breakdown, these structural alterations can lead to the production of smaller, less immunogenic peptides. Numerous processes, such as the Maillard reaction, which forms conjugates with less allergenicity when saccharides and proteins react at high temperatures, might cause these interactions. By interacting with polysaccharides, fermentation processes especially those involving lactic acid bacteria (LAB) can alter protein structures and lessen allergenicity. Furthermore, milk proteins can develop covalent or non-covalent interactions with polysaccharides, which decreases the proteins' capacity to bind IgE antibodies, which are crucial for allergic reactions. It was discovered by Yuan et al. (2025) that the symptoms of cow's milk allergy (CMA) were considerably reduced by chitosan oligosaccharides (COS), demonstrating their potential to reduce milk-protein allergenicity through preventative and intervention measures. A shift toward a more balanced Th1/Th2 immune response and enhanced immunological tolerance to milk proteins was indicated by COS's reduction of allergy-related immunoglobulins (IgE and IgG1) and regulation of important cytokines (e.g., IL-10, IL-17 A, and IFN-γ). Additionally, COS altered the composition of the gut microbiota, including the ratio of Firmicutes to Bacteroidota, which may further lessen allergic response. Early (preventive) delivery had the greatest protective effect, while shorter treatments had less of an impact.

Additionally, Duan et al. (2025) found that covalently conjugating the milk allergen β-lactoglobulin (β-LG) with *Lactiplantibacillus plantarum* exopolysaccharides (EPS) markedly reduced its allergenicity. By forming a β-LG–EPS–(polyphenol) complex, EPS-induced structural changes and surface shielding lowered β-LG–specific IgE binding compared with native β-LG, indicating reduced sensitization potential. This supports polysaccharide conjugation as an effective strategy to mitigate milk-protein allergenicity and develop hypoallergenic dairy ingredients.

## 5 The role of polysaccharides in increase milk protein shelf life

### 5.1 Protection against environmental stressors

Polysaccharides enhance milk protein stability against thermal deterioration and oxidation. They form protective complexes with proteins, shielding them from photo-degradation and UV exposure. This layer also increases thermal stability by preventing heat-induced denaturation and aggregation, improving overall resilience (Gao et al., 2025). Additionally, polysaccharides improve the ability to bind water, maintaining structural integrity during processed at high temperatures. Therefore, a lot of polysaccharides have antioxidant qualities that assist keep proteins from oxidizing when exposed to UV light, maintaining their nutritional and functional value (Wu et al., 2021). According to that Mediwaththe et al. (2023) found that by creating a stabilizing network inside milk protein dispersions with cocoa powder and sugar, κ-carrageenan improves the thermal stability of milk proteins. κ-Carrageenan promoted protein aggregation under combined heat (90 °C/5 min or 121 °C/2.6 min) and shear stress at greater concentrations (0.01–0.05% w/w), leading to larger particles and more viscosity. The instability of micelles and loosely bound caseins within the κ-carrageenan network caused this aggregation, making them more prone to aggregation under shear. Additionally, Lin et al. (2022) demonstrated that coacervation with soy soluble polysaccharides (SSP) plays a crucial role in protecting lactoferrin (LF) from thermal denaturation. The SSP-LF complexes, formed under varying pH (4–7) and SSP ratios (8:1 to 1:16), maintained LF's secondary structure and α-helix content during heat exposure. Through electrostatic interactions, confirmed by Quartz Crystal Microbalance with Dissipation (QCM-D), SSP effectively prevented LF aggregation and preserved its antimicrobial activity post-thermal treatment. On the other hands, Han, Li, et al. (2024) displayed that the antioxidant capacity of emulsions stabilized by whey protein isolate (WPI) is increased by dandelion polysaccharide (DP) and its carboxymethylated derivative (CMDP). The integrity of the emulsion was maintained by the substantial inhibition of oxidation during storage that these polysaccharides provided in the emulsion formulations. By presumably scavenging free radicals and lowering oxidative degradation of the system's proteins and lipids, DP and CMDP successfully increased the emulsions' antioxidant capacity.

### 5.2 Preventing milk protein precipitation

Reducing the risk of milk proteins during food processing, storage, or under specific conditions, “precipitation” typically refers to methods and techniques aimed at preventing milk proteins from aggregating or separating from the solution (Singh et al., 2022). Precipitation usually happens in dairy products when proteins become unstable or when factors (such as temperature, ionic strength, pH, etc.) induce the proteins to cluster and form solid masses, which can alter the product's texture, quality, and appearance (Kapolos et al., 2025). Because polysaccharides stabilize the proteins and stop them from aggregating, they can significantly lower the danger of milk protein precipitation (Markussen et al., 2021). Under some circumstances, such as acidification or heat treatment, milk proteins especially caseins have a propensity to precipitate (Kern et al., 2020). By interacting with the proteins and improving their solubility or by forming a barrier that keeps the proteins from clumping together, polysaccharides can help lessen this. Artemova et al., 2021investigated how apple and beetroot pectin affect the texture of goods with foam and emulsion structures, with a particular focus on how pectin can lower the danger of milk protein precipitation in dairy desserts. Pectin improves the dessert's structural stability by lowering active acidity and raising viscosity, which facilitates the development of protein-polysaccharide complexes with sodium caseinate. By limiting the unwanted precipitation of sodium caseinate proteins, these protein-polysaccharide interactions help to generate stable foam and emulsions. Additionally, Meng et al. (2024) that under certain pH and protein/polysaccharide ratios, carrageenan (CG), xanthan gum (XG), and locust bean gum (LBG) can form lactoferrin (LF)–polysaccharide complexes, improving LF dispersion and stability without causing significant structural disruption. While LBG complexation depended largely on hydrogen bonding/hydrophobic interactions that can provide steric stabilization, CG and XG interacted with LF mostly through electrostatic attraction, boosting charge-based repulsion and perhaps restricting protein aggregation. However, LF–CG and LF–XG displayed precipitation and increased UV absorbance at pH <6, suggesting that precipitation regulation is very pH-dependent and may be weakened in acidic environments.

### 5.3 Microbial modulation

By modifying microbial behavior in two complementary ways suppressing unwanted germs and stabilizing helpful cultures polysaccharides can prolong the shelf life of milk protein-based foods. First, by preventing spoiling and harmful microbes, polysaccharides can shield milk proteins from microbial deterioration. This lowers enzymatic activity and restricts secondary oxidative deterioration (Ben Soltana et al., 2024). Their diverse structures enable antibacterial and antioxidant functions, offering a natural alternative to synthetic additives in preventing enzymatic and oxidative protein breakdown. Several polysaccharides exhibit antibacterial effects through mechanisms such as membrane disruption, particularly against common foodborne pathogens including *E. coli*, *S. aureus*, and L. *monocytogenes*, which supports maintenance of product quality during storage (Ložnjak Švarc et al., 2022). In addition, gum arabic and chitosan have been reported to limit bacterial and fungal growth, helping prevent microbial spoilage and preserve freshness, including through protective coating formation (Felicia et al., 2022). Crucially, in dairy systems, polysaccharides can also promote advantageous microbial stabilization. Polysaccharides can create protective matrices that enhance the survival of probiotic bacteria during processing and storage conditions such drying, heat exposure, and light/UV challenges when mixed with milk proteins (such as whey proteins). By encouraging a favorable microbial balance in functional dairy products, this stabilization supports the antibacterial action of polysaccharides and helps sustain viable probiotic populations and consistent functional performance across shelf life. To support this, Liang et al. (2023) found that at pH 3.0, whey protein concentrate (WPC) and pectin (PEC) produce non-covalent electrostatic complexes that enhance interfacial characteristics and successfully stabilize W/O/W emulsions. The complexes demonstrated improved surface charge and emulsifying efficiency when the PEC concentration was ≥0.8% (*w*/*v*), allowing for effective encapsulation of *Lacticaseibacillus rhamnosus*. When compared to WPC alone, the optimized method produced a high encapsulation efficiency (∼78%) and enhanced probiotic viability under cold storage, simulated pasteurization, and GI digestion.

## 6 The role of polysaccharides in sustainability, waste reduction, and regulatory compliance in food processing

In the food processing sector, polysaccharides are becoming more and more well-known because of their many advantages, which include improving the shelf life and functional qualities of food products as well as lowering waste, promoting sustainability, and satisfying legal requirements. They are perfect candidates to meet the increasing demand for environmentally friendly and compatible food processing methods because of their natural origin, biodegradability, and adaptability (Arshad et al., 2025). Starch, cellulose, and alginate are examples of renewable, biodegradable polysaccharides that provide a sustainable substitute for packaging and artificial additives. Made from food waste or agricultural byproducts, such as seaweed-based alginates, maize starch, or potato starch, they support circular economy models, lessen reliance on petroleum-based products, and lessen environmental damage. Polysaccharides are also utilized in edible coatings and films, which offer a practical substitute for plastic packaging and help cut down on plastic waste. Polysaccharides reduce waste by extending the stability and shelf life of dairy products, particularly milk proteins, by lowering the need for chemical preservatives and preventing spoiling. Additionally, they enhance texture and moisture retention, lowering food waste in perishable goods. Additionally, polysaccharides can encapsulate delicate components like vitamins and probiotics, preventing deterioration and improving nutrient retention across the supply chain (Karnwal et al., 2025). The FDA and EFSA have approved polysaccharides like guar gum, xanthan gum, and carrageenan as safe, natural substitutes for synthetic additives; their use in dairy products guarantees adherence to food safety laws and is in line with clean-label trends, satisfying consumer demand for natural ingredients. The capacity of polysaccharides to substitute petroleum-based packaging materials promotes adherence to environmental and food safety regulations as regulatory agencies place a greater emphasis on sustainability and the reduction of plastic waste (de Oliveira et al., 2025).

## 7 Conclusion and future prospects

The need for food will rise as the world's population is predicted to increase by about 2 billion people by 2050. It will take a lot of teamwork to create resilient food systems and achieve sustainable welfare. Research shows that the per capita demand for dairy products and nutrient-rich milk proteins stays constant as the population increases, even in the face of demographic shifts. Pectin, starches (such as corn starch), gums (such as gum arabic, guar gum, and xanthan gum), konjac flour, and locust bean gum are examples of clean-label polysaccharides that have the potential to improve the functionality of milk proteins, especially when it comes to the creation of environmentally friendly and functional dairy products. Customers are familiar with these polysaccharides, and they meet the growing need for food ingredients to be transparent. According to recent research, polysaccharides can retain milk proteins, enhance their functional qualities, and make it easier to create novel food products that satisfy consumers' increasing need for natural, clean-label ingredients.

Beyond functionality, polysaccharides support sustainability by enabling biodegradable coatings and packaging, reducing dependence on petroleum plastics, extending shelf life, and thereby cutting food waste. They also facilitate valorization of agri-food by-products and improve resource efficiency in formulations. Future research should optimize polysaccharide structure to tune water holding, gelation, and emulsification while maintaining clean-label and low-impact sourcing. Incorporating bioactive polysaccharides with antioxidant or anti-inflammatory potential may meet health-driven consumer demand. Deeper insight into milk protein–polysaccharide interactions could improve nutrient bioaccessibility, reduce allergenicity, and enhance overall health value, aligning with the shift toward transparent, sustainable, personalized dairy systems for future product innovation.

## 8 Author's perspective

From our perspective, the most promising direction is to move beyond demonstrating functionality toward designing predictable milk protein–polysaccharide systems by linking polysaccharide structure (charge density, molecular weight, branching, and conformation) with targeted outcomes such as water holding, gel strength, emulsion stability, and sensory quality. We also believe that standardized characterization methods and pilot-scale validation are essential to translate laboratory findings into industrial applications. Finally, integrating clean-label polysaccharides with sustainable sourcing (including upcycled by-products) and health-oriented functions offers a practical pathway to develop next-generation dairy products that are stable, consumer-acceptable, and environmentally responsible.

## CRediT authorship contribution statement

**Mohamed Aamer Abubaker:** Writing – original draft. **Duoduo Zhang:** Investigation. **Haorui Ma:** Investigation. **Runru Xiang:** Methodology. **Yiran Wang:** Investigation. **Abdalla Musa Elimam:** Investigation. **Majida Al-Wraikat:** Writing – review & editing. **Yongfeng Liu:** Project administration.

## Funding

This work was financially supported by Shaanxi Science and Technology Plan Projects of China (2024NC-ZDCYL-03-07, 2024NC-GJHX-30), Shaanxi Province Market Supervision Bureau Science and Technology Plan Projects of China (2024KY25), Xi'an City Science and Technology Plan Projects of China (24NYGG0018), the Fundamental Research Funds for the Central Universities in China (GK202406041, GK202406040, GK202306005).

## Declaration of competing interest

The authors declare that they have no known competing financial interests or personal relationships that could have appeared to influence the work reported in this paper.

## Data Availability

No data was used for the research described in the article.
